# Generation and plant production of recombinant fluorescent immunoglobulin G as innovative immunodiagnostic reagents

**DOI:** 10.1111/pbi.70235

**Published:** 2025-07-01

**Authors:** Ramona Sterpa, Marcello Catellani, Patrizia Melpignano, Thea Serra, Laura Anfossi, Cristina Capodicasa

**Affiliations:** ^1^ Laboratory Green Biotechnology ENEA, Casaccia Research Center Rome Italy; ^2^ OR‐EL d.o.o. Kobarid Slovenia; ^3^ Department of Chemistry University of Turin Turin Italy

**Keywords:** fluorescent proteins, plant molecular farming, recombinant antibodies, LFIA, OLED‐based sensor, *Nicotiana benthamiana*

## Abstract

Immunodiagnostic systems for on‐site screening are increasingly in demand but require expensive reagents such as chemically modified antibodies. To obtain biomolecules as ready‐to‐use reagents for diagnostics, new recombinant fluorescent immunoglobulins G were generated by fusion of the heavy chain of a monoclonal antibody, 5H3, to two different fluorescent proteins: Green Fluorescent Protein (5H3GFP) and Cyan‐excitable Orange Fluorescent Protein (5H3CyOFP1). The engineered antibodies were expressed by transient agroinfiltration in *Nicotiana benthamiana* plants, used as bioreactor sustainable, cost‐effective and easy to scale up, with a yield of 80 and 35 mg/kg of purified 5H3GFP and 5H3CyOFP1, respectively. Both recombinant proteins retained the ability to recognize the antigen of the original mAb (aflatoxin M1, a contaminant of dairy products), while simultaneously being able to emit fluorescence in the green or orange light. To demonstrate their practical application in the diagnostic field, fluorescent antibodies were exploited to set up two fast screening systems for aflatoxin M1 detection: a lateral flow immunoassay and an OLED‐based device. For both diagnostic systems, antigen‐specific fluorescence signals as well as a first competitive assay were obtained. Although these assays will need a further implementation, our results demonstrate a concrete possibility to develop novel and fast analytical systems based on fluorescent recombinant antibodies produced in plants.

## Introduction

Diagnostic analyses, both in biomedical and agri‐food fields, have become increasingly numerous and important over the years. In addition to analytical methods, such as HPLC (High Performance Liquid Chromatography) or MS (Mass spectrometry), mainly used for greater accuracy of the quantitative data (Hameedat *et al*., 2022), on the market there are many screening kits based on immunodiagnostic techniques, such as ELISA (Enzyme Linked ImmunoSorbent Assay) (Liu *et al*., 2023). Even if the ELISA is a good compromise between analytical accuracy and execution rapidity, novel systems that are faster, cheaper, portable and easy to use for on‐site screening are still needed. Currently, the most used portable diagnostic system worldwide is the lateral flow immunoassay (LFIA) that allows one to perform an analysis on the field in a simple, fast and economic way. However, one drawback of this method is its limited sensitivity. Usually, the results reading is based on optical detection (visually observed for a qualitative assessment or quantified by acquiring and processing images of the devices) (Park, 2022). Nevertheless, colorimetric detection has been associated with poor sensitivity and the use of fluorescent probes has been deeply investigated in recent years to solve the problem. In an early stage, fluorescent organic dyes were employed for developing fluorescence LFIA (FLFIA); however, due to the limited photostability and low quantum yield (QY), they were replaced by fluorescent nanoparticles (Gong *et al*., 2017). These nanostructured materials allowed for improving the sensitivity of FLFIA; however, they were poorly compatible with the aqueous environment of FLFIA typical applications or suffered from the complicated preparation and use of toxic and/or rare elements, which increased costs (Bruno, 2022; Seo *et al*., 2023). Furthermore, in the last decade, new point‐of‐care systems have been developed that foster the potential of fluorescence detection to efficiently improve the assay sensitivity. Among these innovative point‐of‐care diagnostic systems, an OLED‐based reader has been developed to allow the detection of different biomolecules (like toxins, antibodies, etc.) in a liquid matrix with a very high sensitivity and without any amplification step. This point‐of‐care system needs specific antibodies conjugated with appropriate fluorophores that meet particular spectral characteristics. An absorption spectrum overlapping the OLED spectral emission and a large Stokes shift are important to optimize the detection sensitivity, as well as a high molar absorption coefficient and a high fluorescence QY (Melpignano *et al*., 2014; Pope and Swenberg, 1999). Several commercial dyes satisfy these requirements; however, to use these dyes in a diagnostic assay, a time‐consuming and not always efficient and economic process of antibodies conjugation is necessary. Furthermore, the chemical conjugation of dyes may cause reproducibility issues, such as alterations in antigen binding or antibody properties (Joubert *et al*., 2019; Shrestha *et al*., 2012; Vira *et al*., 2010).

In this work, antibodies intended to develop immunodiagnostic devices were marked through a biotechnological approach: fusing them directly to fluorescent proteins. Fluorescent proteins are valuable tools for *in vivo* imaging of cells and tissues and a variety of other applications. The first fluorescent protein studied and cloned is the green fluorescent protein, GFP, from the jellyfish *Aequorea victoria* that absorbs ultraviolet light, emitting green light (Shimomura, 2009). Since the discovery of avGFP, many fluorescent proteins are now known and expressed as recombinants, both isolated from different organisms and obtained by mutagenesis of the original protein. The protein mGFP5ER (excitation‐emission spectrum 395 nm–509 nm), for example, derives from the avGFP, whose gene sequence has been optimized for expression in plants (Haseloff *et al*., 1997). The orange‐red fluorescent protein CyOFP1 instead has the chromophore like that of DsRed present in corals, with excitation and emission peaks at 497 nm and 589 nm, respectively (Chu *et al*., 2016). This protein was obtained by mutagenesis of the mNeptune2 red protein sequence. The modifications give the CyOFP1 unique characteristics: a much higher luminosity than its red fluorescent predecessors, high photostability, a long half‐life of fluorescence and a rapid maturation time of the chromophore. Furthermore, the absorbance peak of CyOFP1 is shifted by about 100 nm with respect to its predecessor, thus in the blue (cyan) region.

The idea of obtaining fluorescent antibodies biotechnologically has already been explored in the past; engineered antibodies have been expressed mainly in bacteria and also in plant cells as single‐chain fragments (scFv) (Knödler *et al*., 2023; Markiv *et al*., 2011; Morino *et al*., 2001). This antibody format, the simplest to express, can be useful for certain applications but is not comparable to a complete immunoglobulin, which is more stable and has a higher affinity (avidity) to the antigen required for high sensitivity of a diagnostic kit. Fluorescent IgG was expressed in *Escherichia coli* and mammalian cells (Haas *et al*., 2010). In *E. coli*, however, heavy and light chains were expressed separately in cytoplasmic inclusion bodies, requiring a laborious process of denaturation, mixing of light and heavy chains and refolding of IgG (Luria *et al*., 2012). Fluorescent heavy and light chains of a monoclonal antibody have also been expressed in protoplast and epidermal cells of tobacco to elucidate the trafficking of pharma‐proteins through living plant cells (Irons *et al*., 2008). In this study (as in most articles in the literature) fluorescence was mainly used to follow recombinant protein addressing or accumulation *in vivo*; therefore, yield, purification and effective characterization of the antibodies have not been reported. Here we produced in *Nicotiana benthamiana* plants, used as bioreactor, fluorescent recombinant antibodies exploited as novel reagents for developing rapid immunodiagnostic assays for the detection of aflatoxin M1. Aflatoxin M1 (AFM1) belongs to the class of mycotoxins, products of the secondary metabolism of toxigenic fungi that can contaminate foodstuffs such as cereals, legumes and dried fruit, both in the field and after harvesting. In relation to the risk deriving from the intake of mycotoxins, their quantities are strictly regulated by the European Community, which has set maximum levels for AFM1 in dairy products of 50 ng/kg for adults and 25 ng/kg for infants (Commision of the European Communities, 2006). A specific antibody for AFM1, mAb 5H3, exploited to set up a rapid competitive ELISA assay for the quantification of the toxin in milk, was recently described (Capodicasa *et al*., 2022). The high affinity of this mAb allowed us to develop a diagnostic assay able to respond to the limits of the EU law, with an IC50 < 25 ng/L and a quantification range between 5 and 150 ng/L.

Starting from the mAb 5H3, in this work two recombinant and fluorescent antibodies were obtained by gene engineering and produced in plants, using a transient expression system mediated by *Agrobacterium tumefaciens* (Vaquero *et al*., 1999). The production of biopharmaceutical proteins in plants is called Plant Molecular Farming (PMF). PMF is considered a technology that has grown and made tremendous progress in the last two decades. The development and improvement of transient expression system has significantly reduced the protein production time and greatly improved the protein yield in plants. The main advantages of PMF over conventional expression system (mammalian cell cultures) are, scalability, flexibility, versatility, sustainability and cost‐effectiveness of platforms needed for proteins production (Buyel, 2019; Schillberg and Finnern, 2021). Conversely, one disadvantage is represented by the high cost of downstream processing of a biopharmaceutical molecule, although this, in the case of a diagnostic reagent, could be reduced. The recombinant fluorescent antibodies (Abs) obtained in plants served as ready‐to‐use reagents for the development of rapid assays such as LFIA and an OLED‐based biosensor.

## Results and discussion

### Transient plant expression of a monoclonal antibody fused to GFP and CyOFP1

**Figure 1:**
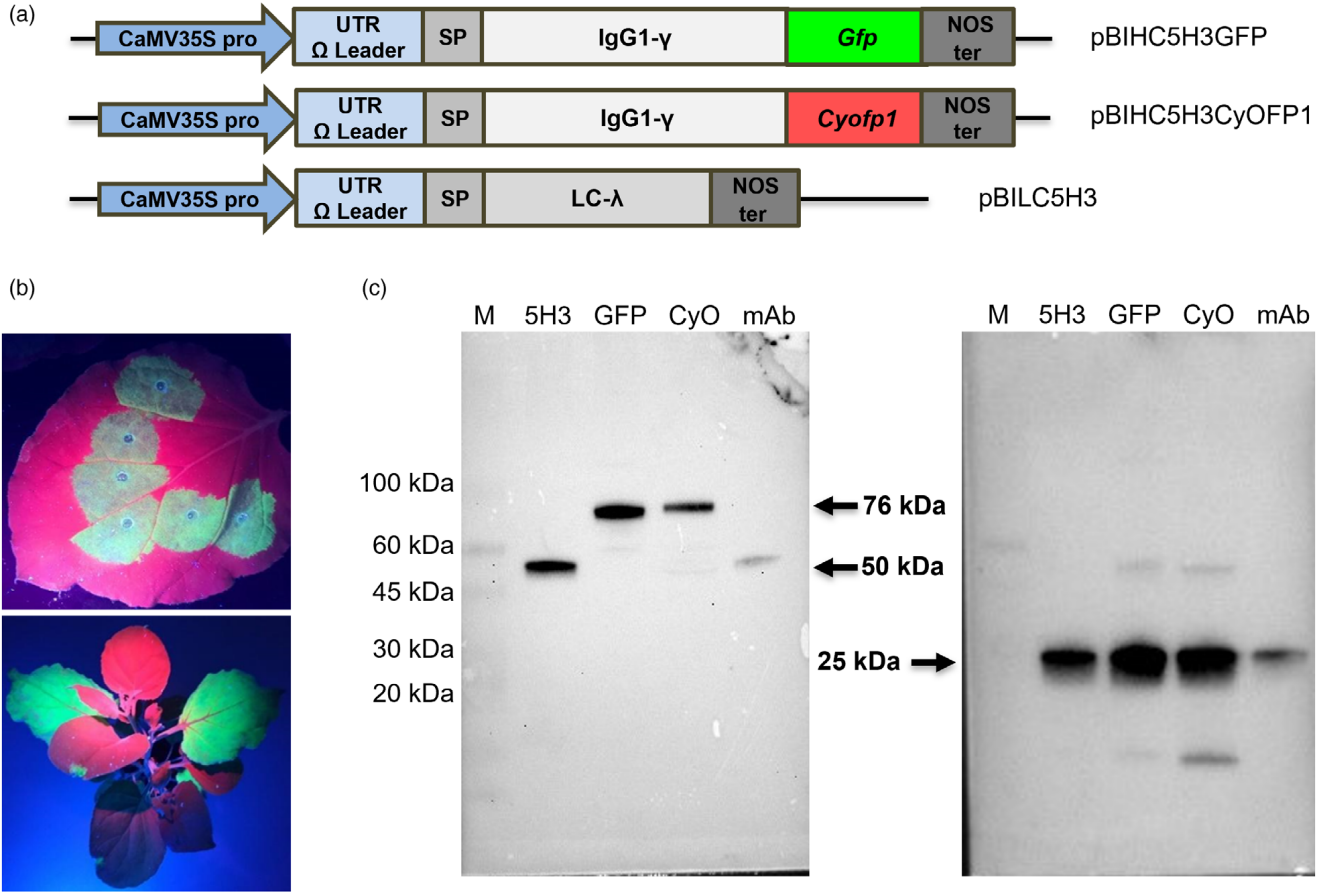
Expression in plants of fluorescent mAbs. (a) Schematic representation of constructs for plant expression of mAb 5H3 fused to fluorescent proteins. pBIHC5H3GFP or CyOFP1: vector containing the heavy chain of a mouse IgG1 fused to GFP or CyOFP1, respectively, encoding sequence. pBILC5H3: vector containing the light chain (lambda) encoding sequence. All genes were under the control of the CaMV 35S promoter and Omega translation enhancer. (b) Fluorescence of agroinfiltrated *Nicotiana benthamiana* plants expressing mAb 5H3GFP under UV light. (c) Western blot analysis in reducing conditions of TSP extracted from leaves of plants infiltrated with agrobacteria containing pBIHC5H3 and pBILC5H3 vectors for 5H3 IgG (5H3), pBIHC5H3GFP and pBILC5H3 vectors for 5H3GFP IgG(GFP), pBIHC5H3CyOFP1 and pBILC5H3 vectors for 5H3CyoFP1IgG(CyO). Purified 5H3 mAb (mAb) was used as a positive control. Polyclonal anti‐mouse IgG or anti‐mouse lambda antibodies were used to detect the heavy chains (left) and light chains (right), respectively.

To obtain a fluorescent full IgG starting from the mAb 5H3 (Capodicasa *et al*., 2022), the sequence encoding the heavy chain of the antibody was fused with that encoding a green fluorescent protein (GFP), or an orange‐red fluorescent protein (CyOFP1). In particular, the coding sequence of both fluorescent proteins optimized for expression in plants was fused at the C‐terminus of the heavy chain while the light chain of 5H3 was not changed (Figure 1a). As fluorescent proteins, GFP was chosen because it is the most frequently used and the version already optimized for expression in plants (Haseloff *et al*., 1997) was available, while CyOFP1 has a wider excitation and emission spectrum in the visible and a higher brightness (Chu *et al*., 2016), optimal properties for diagnostic systems (especially for OLED‐based device) further developed. The full IgGs, 5H3GFP and 5H3CyOFP1, were expressed in *N. benthamiana* plants by agroinfiltration. For 5H3GFP, it was possible to follow the expression of recombinant protein fused to GFP starting from 3 to 4 days post‐infiltration (dpi), observing a green fluorescence in the infiltrated tissues illuminated with UV light (365 nm) (Figure 1b). In the case of 5H3CyOFP1 instead it was not possible to monitor the expression and see an evident specific fluorescence illuminating agroinfiltrated leaves with blue light, likely due to an overlapping of autofluorescence of plants with that of CyOFP1. Chlorophyll is excited by UV, blue or green light and emits strongly in the red with an emission at 600–800 nm, in addition the carotenoid emission at 500–550 nm is very close with that of CyOFP1 (Donaldson, 2020). This suggests that CyOFP1 cannot be used as an *in vivo* reporter in plants by directly illuminating with blue light or, at least for this purpose, suitable filters must be used. The expression of recombinant Abs was evaluated by Western blot analysis in reducing condition of total soluble proteins (TSP) of extracts from agroinfiltrated leaf disks (sampled at 6 dpi). The analysis confirmed the successful co‐expression of unmodified light chain and both recombinant heavy chains, showing (Figure 1c) a band corresponding to the expected molecular weight for the γ heavy chain fused to the fluorescent proteins GFP and CyOFP1 (77 kDa and 76 kDa, respectively), higher than that of the unmodified heavy chain (50 kDa) of mAb 5H3. Moreover, the accumulation of fluorescent mAbs in agroinfiltrated leaves starting from the 4^th^ up to the 7^th^ dpi was evaluated by ELISA and summarized in Table 1. Both antibodies were successfully expressed in *N. benthamiana* plants, using the agroinfiltration technique, with a similar trend for the two antibodies showing a peak of accumulation at 6–7 dpi. In terms of yield, however, the antibodies fused to fluorescent proteins showed a lower accumulation than the original antibody and the 5H3CyOFP1 had the lowest yield even compared to the 5H3GFP (Table 1).

**Table 1:** Yields of recombinant Abs in agroinfiltrated leaves of *N. benthamiana*

Recombinant mAb	Yields (mg/kg)			
	4 d.p.i.	5 d.p.i.	6 d.p.i.	7 d.p.i.
5H3	669.6 ± 184.9	1035.7 ± 214.3	1581.0 ± 153.1	1852.8 ± 163.2
5H3‐GFP	532.9 ± 205.7	639.1 ± 191.3	1001.9 ± 280.5	1160.0 ± 261.3
5H3‐CyOFP1	186.8 ± 41.4	268.7 ± 45.2	489.8 ± 179.3	450.8 ± 152.5

Yields of recombinant Abs in the leave extracts from three independent agroinfiltrations were calculated by quantitative ELISA and values indicate average yield ± standard error (SE, *n* = 3). The yields are reported as Ab mg for kg of fresh weight (FW) in total soluble proteins (TSP) extracts of agroinfiltrated leaves.

Since the gene construct for light chain expression was the same for all IgGs, the lower yield of fluorescent Abs seemed due to a lower expression of modified heavy chain. This result was not so surprising since this is an artificial protein that could be less efficiently synthesized compared to the original heavy chain or in part eliminated. Some fusion proteins, in fact, could fold incorrectly and the misfolded proteins were eliminated by proteasomes. Less clear was the lower expression level of the heavy chain fused to CyOFP1 compared to that fused to GFP and to improve the yield of 5H3CyOFP1, a second recombinant antibody was constructed by inserting a sequence for protein retention in the endoplasmic reticulum (ER) *K‐DEL* (Lys‐Asp‐Glu‐Leu) at the C‐terminus of CyOFP1. This sequence has a stabilizing function for proteins since the ER contains few proteases responsible for protein degradation and a large quantity of chaperonins that support proteins in their correct folding. Furthermore, the maintenance of secreted proteins within the ER improves their accumulation compared to targeting them in the secretory pathway (Conrad and Fiedler, 1998). Following the transient expression in plants, the quality and expression levels of this second recombinant antibody were verified by Western blot and DAS ELISA (Data not shown). However, despite the presence of the tetrapeptide *K‐DEL*, the expression levels and yield of the 5H3CyOFP1ER antibody also did not appear to be positively affected, suggesting a lower accumulation due to the intrinsic characteristics of the fluorescent protein rather than to degradation phenomena that can be prevented by the permanence in the ER. CyOFP1, unlike GFP, is an artificial protein deriving from a very extensive mutagenesis which may have affected its stability. While GFP is known to accumulate in good quantities in many cellular systems and also in plants (Mardanova and Ravin, 2021; Yamamoto *et al*., 2018), no data on the CyOFP1 expression levels are reported in the few works present in the literature in which the protein has been expressed in *E. coli* as a reporter (Nuñez *et al*., 2017; Subach *et al*., 2021). Another possibility could be that in fusion with the heavy chain, the CyOFP1 folded incorrectly with respect to GFP, resulting in a minor accumulation of recombinant protein.

In the future, a strategy to increase the yield of recombinant IgG, and in particular of 5H3CyOFP1, could be the fusion of a fluorescent protein to the heavy chain through different linkers or to the light chain of IgG, as demonstrated by the work of Haas *et al*., 2010, in which the fluorescent protein Citrin was fused to antibodies in various configurations, reporting that different fusions influenced the expression levels obtained.

### Purification and characterization of recombinant fluorescent mAbs

The recombinant antibodies were purified by protein G affinity chromatography from leaves of vacuum‐agroinfiltrated plants harvested 6 days post‐infiltration, following the various steps by illuminating the column and the fractions with UV light (Figure S1).

The average purification yields obtained were about 80 and 35 mg/kg for 5H3GFP and 5H3CyOFP1, respectively, with a recovery of about 65% and a purity >90%. The different yield reflected the different expression levels obtained for the engineered Abs but were in line with those previously reported for IgG expressed in plants (Frigerio *et al*., 2022; Jugler *et al*., 2022). In addition, a little deviation from standard recovery obtained purifying mAb 5H3 (~75%) was observed suggesting that the biotechnological fusion slightly affected the binding to protein G, although only in terms of purification efficiency and not the final purity level of the fluorescent mAbs.

The SDS‐PAGE analysis of Abs purification in reducing condition confirmed the presence of predominant bands corresponding to the light chain (25 kDa) and to the heavy chain fused to the fluorescent protein (~75 kDa) for both Abs. For 5H3CyOFP1, however, there were additional bands (a main band of about 50 kDa) that indicated a fragmentation of the recombinant heavy chain.

**Figure 2:**
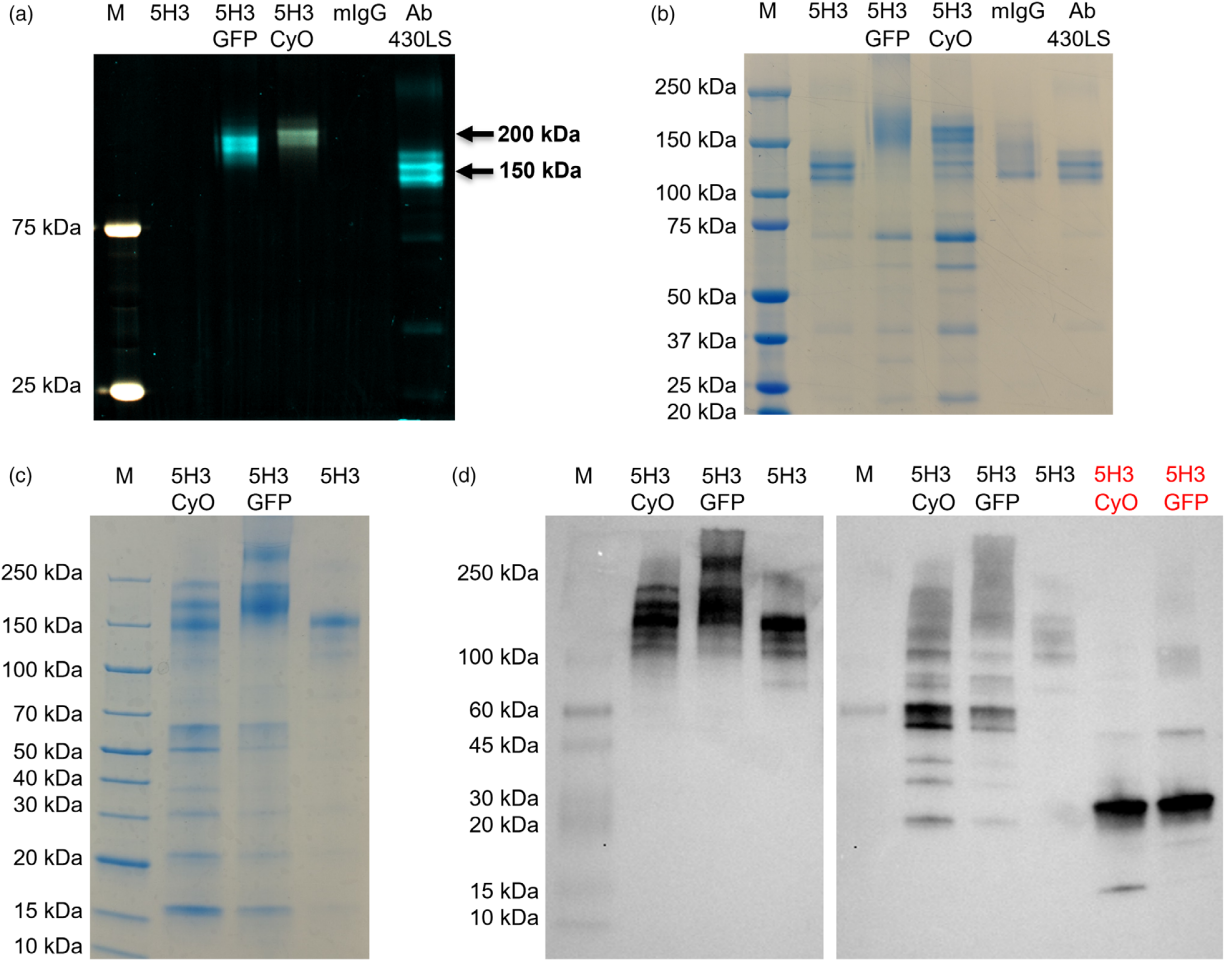
Purified fluorescent mAbs characterization. Purified mAbs were resolved on a 4%–12% gradient gel (SDS‐PAGE) in non‐reducing conditions and mild denaturing conditions (no samples boiling), to preserve fluorescence emission. Fluorescent signals were acquired by the iBright imager (exc = 490–520 nm, em = 568–617 nm) (a) and the same gel was stained by Coomassie after acquisition (b). SDS‐PAGE Coomassie stained (c) and Western blot analysis (d) in standard non‐reducing condition (except for the two reduced samples indicated with red characters) of purified recombinant Abs. The WB membrane was incubated with polyclonal anti‐mouse IgG to detect the heavy chain of IgG (left) or anti‐mouse lambda (right) antibodies to detect the light chain of IgG. Samples loaded: Precision Plus Protein™ Dual Colour Standards (Bio‐Rad) (M), 5H3 IgG (5H3), IgG5H3 fused to GFP (5H3GFP), IgG 5H3 fused to CyOFP1 (5H3CyOFP1), mouse IgGs (mIgG) and IgG labelled with fluorescent dye ATTO 430LS (Ab 430LS).

The purified antibodies were characterized also by non‐reducing SDS‐PAGE analysis that confirmed the assembly and fluorescence of recombinant immunoglobulins. As shown in the SDS‐PAGE analysis performed in mild denaturing conditions (Figure 2a) with a fluorescence imaging system, for both recombinant mAbs, bands significantly higher than that of the IgG used as a control (chemically conjugated to a fluorescent dye) and corresponding to the expected molecular weight for both 5H3GFP and 5H3CyOFP1 (about 200 kDa) were detected. This result confirmed the assembly of the full IgGs conjugated with both fluorescent proteins, confirming also their correct folding and functionality. After Coomassie staining of the same gel (Figure 2b), additional bands of lower molecular weight, more consistent for 5H3CyOFP1, were also detected, suggesting a partial degradation of antibodies. In several studies in which antibodies were expressed in plants, the presence of protein fragments between 20 and 150 kDa was highlighted in SDS‐PAGE analysis under non‐reducing conditions, indicating a degradation of the antibody. These fragments can be attributed to proteolytic processes that take place in the apoplast that is a cellular compartment where proteolytic enzymes reside and where the antibodies expressed in plants were directed by the secretory pathway, necessary to obtain post‐translational modifications such as glycosylation and disulfide bridges (Hehle *et al*., 2015; Jutras *et al*., 2020). In addition, or alternatively, to products of proteolysis, some of these bands seemed to indicate the presence of light chain dimers, as evidenced by the WB analysis carried out on the purified antibodies in non‐reducing conditions. In this analysis (Figure 2c,d), in fact, some bands around 50–60 kDa were recognized by the anti‐mouse lambda polyclonal antibodies and not by the anti‐mouse IgG ones. Moreover, in the SDS‐PAGE analysis performed in mild conditions that allowed detection of fluorescent signals (Figure 2a), as already reported, only fluorescent bands corresponding to complete IgGs and no fluorescent fragments of lower molecular weight were detected. This could be explained by an accumulation in the plant cells of light chain that is able to dimerize through disulfuric bridges, due to the unavailability of sufficient quantities of the protein partner, the heavy chain, to assemble in full IgG. This accumulation appeared to be inversely proportional to the amount of complete immunoglobulin obtained: 5H3CyOFP1 > 5H3GFP > 5H3.

In addition, a gel filtration analysis was carried out to establish the molecular weight of fragments and full antibodies (Figure S2). Size‐exclusion chromatography on a Superdex™ 75 10/300 GL column revealed a major protein fraction corresponding to the fully assembled IgG for both purified Abs: peak eluting at 7.71 or 7.93 mL for 5H3GFP and 5H3CyOFP1 respectively, compared to the peak eluting at 8.04 mL of the mouse IgG used as a control. Other two fractions (peaks eluting at 9.82 and at 11.18 for 5H3CyOFP1 and 9.87 and 11.13 for 5H3GFP), corresponding to antibody portions with an estimated molecular mass of about 56 kDa and about 28 kDa respectively, were observed for both antibodies, although in a much smaller percentage for 5H3GFP. This observation, as already described above, is consistent with the molecular weights of a lambda light chain and its dimer.

**Figure 3:**
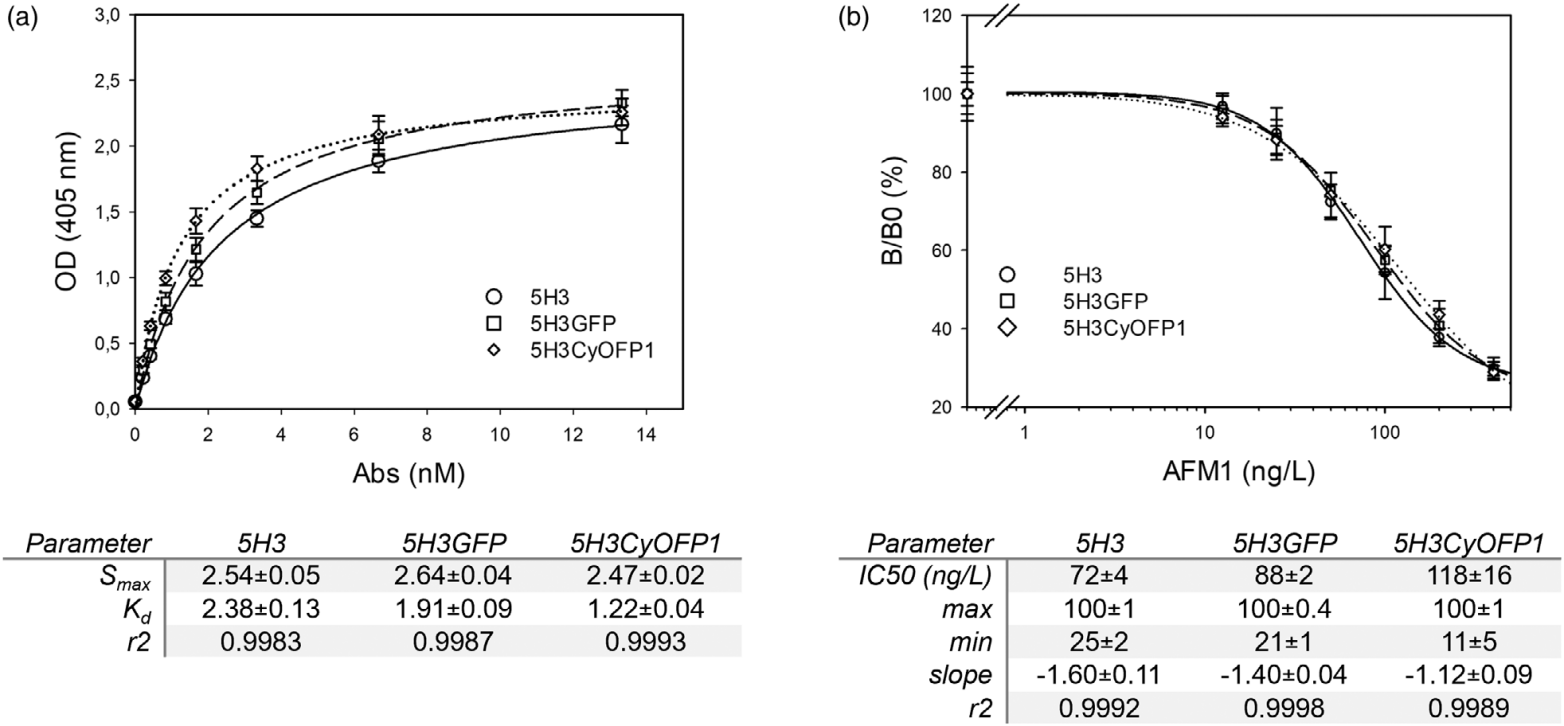
Antigen binding properties of fluorescent antibodies. Binding curves and relative parameters of the 5H3‐CyOFP1, 5H3GFP and the original 5H3 antibody to antigen AFM1 by ELISA (a). Different concentrations of Abs (from 13.33 to 0.21 nM) on AFM1‐BSA coated wells were tested. K_d_ is the apparent dissociation constant, and Smax represents the signal at the saturation of binding sites. (b) Indirect competitive ELISA inhibition curves obtained for the different antibodies for competition with quantities of aflatoxin M1 between 12.5 and 400 ng/L. Parameters obtained by 4PL equation fitting of competition curves. Max and min: values of the lower and upper asymptotes; slope: slope at inflection point; IC50: analyte concentration that inhibited the maximum signal at 50%. Statistical comparison of IC50 values was performed by the one‐way ANOVA (α = 0.05). Values represent average ± SD (*n* = 2).

The functionality of purified recombinant antibodies was verified by ELISA. First, the binding of the fluorescent Abs to the antigen, aflatoxin M1, were evaluated and compared to that of the original mAb 5H3, described and characterized in Capodicasa *et al*. (2022) (Figure 3a). Binding curves showed a similar ability of the new fluorescent mAbs to bind to the antigen. Especially, the 5H3CyOFP1 showed a lowered apparent dissociation constant, which may indicate an increased affinity towards the immobilized antigen.

Finally, an indirect competitive ELISA was used to verify that the recombinant antibodies maintained the same ability as mAb 5H3 to compete with free Aflatoxin M1. A preliminary titration assay was carried out to obtain equimolarity for the original mAb (~150 kDa) and the fluorescent proteins conjugated mAbs (~200 kDa) in the competitive assay. Figure 3b shows the curves obtained from the indirect competitive ELISA. According to the One‐Way ANOVA (α = 0.05) (McDonald, 2014; Ross and Willson, 2017), there was a statistically significant difference between IC50 values of the three Abs (*P* = 0.003, power of performed test 0.983). Using the Holm‐Sidak method to compare the fluorescent antibodies to 5H3 as the control mAb, the difference was statistically significant only for 5H3CyOFP1 (*P* = 0.02), while 5H3GFP was not statistically different from 5H3 (Haynes, 2013; Šidák, 1967). IC50 values slightly increased for the fluorescent mAbs compared to the unmodified 5H3; however, they were in the same order of magnitude, and the difference was statistically significant only for 5H3CyOFP1 (although limited). The curve obtained for these mAbs also showed a lower background signal (min, predicted lower B/B_0_% value), which expanded the dynamic range of the curve. Overall, these data suggest that the biotechnological fusion of the Ab heavy chain with the fluorescent proteins does not interfere with the binding affinity of the Abs with the antigen. Other works have reported C‐terminus biotechnological fusions between Abs heavy and light chains with different proteins of interest, confirming Abs ability to keep their specificity for the target (Haas *et al*., 2010; Silver *et al*., 2021).

### Development of diagnostic devices to detect the aflatoxin M1 exploiting mAbs fused to GFP and CyOFP1

#### LFIA

**Figure 4:**
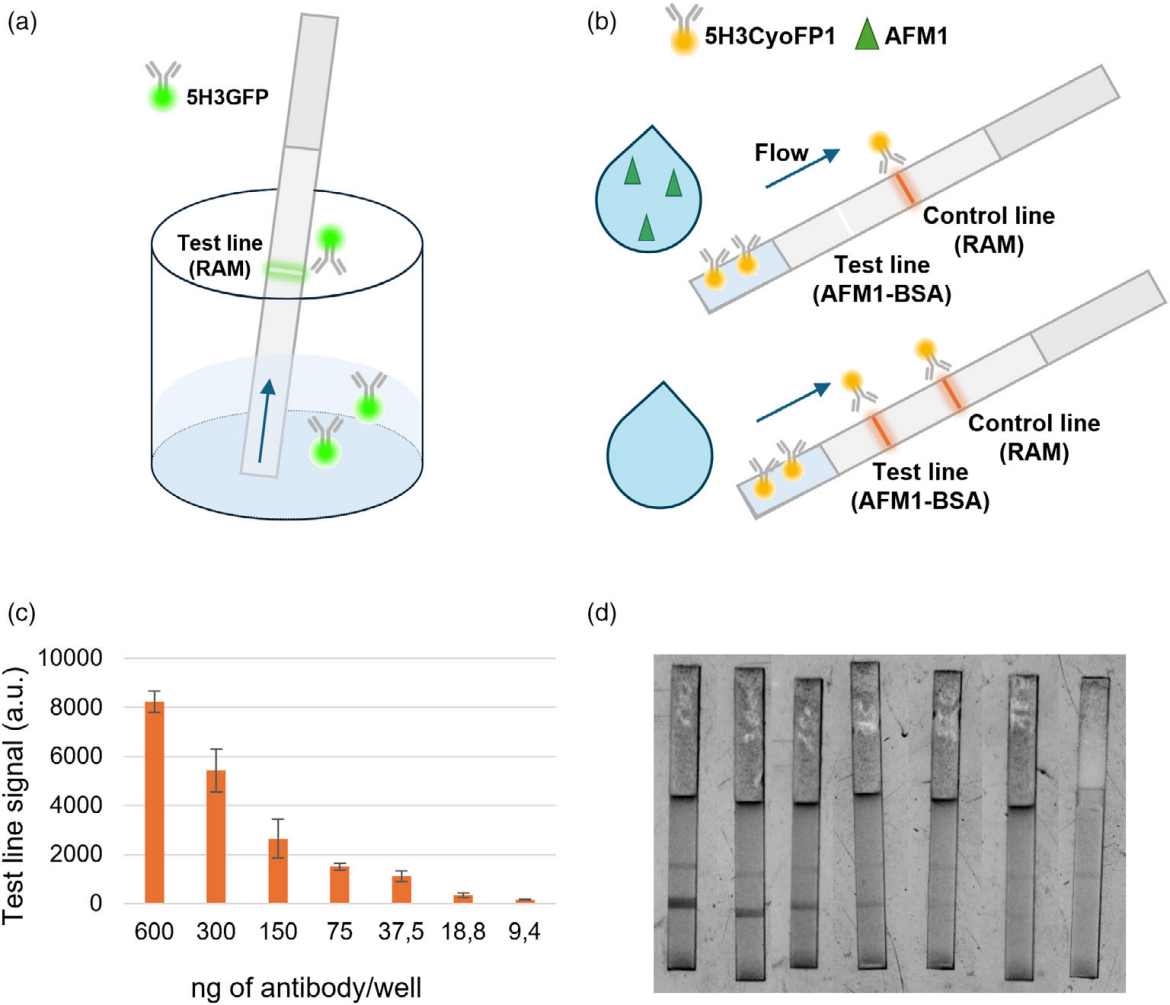
LFIA setup. (a) scheme of the LFIA in the dipstick format used to define buffer composition: the strip functionalized with a line of RAM was immersed in the buffer containing the fluorescent mAb, which flowed by capillarity and bound to the reactive line. (b) Scheme of LFIA strip containing the AFM1‐BSA and the RAM antibodies to form the test and control lines respectively; during the flow, the fluorescent mAb reacts with the AFM1 (if present in the sample) and the binding to the AFM1‐BSA test line is inhibited. In the absence of AFM1, the fluorescent mAb binds to the test line and produces a fluorescent signal. The fluorescent mAb is also captured by the RAM, irrespective of the presence of the analyte, so that the fluorescence is always observable at the control line. Luminescence signals (c) and images (d) provided by the LFIA including 5H3CyOFP1 as the probe as a function of the dilution of the recombinant fluorescent mAb. Images were acquired using the iBright FL1500 (exc = 490–520 nm em = 568–617 nm) and were analysed using the ImageJ processing software to quantify the emitted light. Values represent average ± SD (*n* = 2).

The possibility of employing the new fluorescent antibodies as probe for LFIA was investigated. Preferred characteristics of probes to be employed in LFIA are: (i) providing an intense and reproducible signal, which can be easily detected when accumulated at the test line, (ii) full compatibility with the materials used to build LFIA devices (e.g. nitrocellulose, glass and cellulose pads, etc), (iii) long‐term stability in dry form and fast and efficient re‐suspension upon application of the sample and (iv) low (or absent) non‐specific binding (Di Nardo *et al*., 2021; Kim *et al*., 2023; Li *et al*., 2023). To verify these characteristics, a preliminary study with 5H3GFP was carried out on a model non‐competitive assay. Briefly, a secondary antibody (anti‐mouse IgG produced in rabbit, RAM) was dispensed onto the nitrocellulose (NC) membrane to form the test line, and an adsorbent pad was added to the end of the strip, to facilitate the flow. The new fluorescent antibody was diluted in a microwell and let to flow by capillarity along the membrane (Figure 4a).

Several buffers were examined for their ability to enable the probe flowing along the detection membranes and interacting with the RAM. In detail, we varied buffer pH and additives (various amounts of BSA and Tween20 were considered). The best composition of the dilution buffer was evaluated according to the formation of the complex between the RAM and 5H3GFP, which was revealed by an intense fluorescence signal at the test line, and to the absence of background noise, which was considered assessing the complete transfer of the probe through the nitrocellulose membrane towards the absorbent pad (Figure S3a). The selected buffer was Tris–HCl 20 mM pH 8.5 supplemented with 0.5% Tween 20 and 1% BSA. We confirmed that the fused protein retained the ability of emitting light after flowing across the membrane and being captured by the anti‐mAb antibody anchored onto it. The fluorescence was proportional to the amount of the 5H3GFP (Figure S3b) and was only partially affected by the presence of increasing quantity of NaCl (Figure S3c) and by varying the buffer pH (Figure S3d). These results opened the employment of the fused protein as a new LFIA fluorescent probe. Therefore, we designed a LFIA for revealing AFM1, the antigen recognized by mAb 5H3, based on the competitive immunoassay format. Then, the test line comprised the antigen (AFM1‐BSA), which competed with the AFM1 for the binding to the recombinant fluorescent mAb (Figure 4b). Accordingly, when no AFM1 was present, the mAb interacted with the antigen, ultimately providing a fluorescence signal at the test line. On the contrary, the addition of AFM1 saturated the fluorescent mAb, which was not able to interact with the anchored antigen resulting in a signal decrease at the test line. Unfortunately, the NC membrane with AFM1‐BSA dispensed showed a significant background noise when excited with the 365 nm light to reveal 5H3GFP, thus making difficult to distinguish any specific signals (data not shown). For this reason, subsequent experiments were carried out using 5H3CyOFP1 only.

In the absence of AFM1 added, the 5H3CyOFP1 probe showed an intense fluorescent signal at the test line and an exponential decay of the emitted light as a function of the decrease of the probe amount. The signal disappeared completely when the amount was lowered below 9.38 ng/strip (Figure 4c,d). Measurements repeated on the same day and on different days showed high reproducibility (Table S1). The assay based on the 5H3CyOFP1 was affected by both the ionic strength and pH of the buffer. In detail, the signal intensity of the test line decreased with acidic pH and with increasing ionic strength. We observed a decrease of about 25% in signal loss when the pH decreased from 8 to 5 and an even sharper decrease of about 60% in signal loss for high salt concentrations (Figure S4). Nevertheless, in agreement with the assay format, the addition of AFM1 inhibited the binding of 5H3CyOFP1 to the test line (Figure S5). Despite the level of the mycotoxin used in the experiment being far higher than those of regulatory interest, nonetheless, it confirmed that the new fluorescent probes, and especially the 5H3CyOFP1, were suitable for developing fluorescence LFIA.

#### OLED‐based diagnostic device

The two fluorescent antibodies, 5H3GFP and 5H3CyOFP1, were also tested for use in an OLED‐based device. The fluorescence signals of the mAb fused to GFP or CyOFP1 were initially tested using the standard OLED installed in the portable reader developed at OR‐EL d.o.o (Marcello *et al*., 2013). The spectral emission of this OLED, with a peak wavelength at 434 nm, is not perfectly optimized for these two fluorescent proteins, however there is an acceptable overlap of the spectral emission of the OLED and the spectral absorbance of the GFP and CyOFP1, which allows to excite their fluorescence and test both using the same optical source. In the figure S6 the overlapping of the normalized OLED spectral emission with the GFP and CyOFP1 spectral absorption is shown. The better overlap is obtained with the CyOFP1, which also presents a higher molar absorption coefficient (extinction coefficient) and fluorescence quantum yield (Q.Y. = 0.76, E.C. = 40 000) than the GFP (Q.Y. = 0.79, E.C. = 27 600), with an expected higher brilliance.

**Figure 5:**
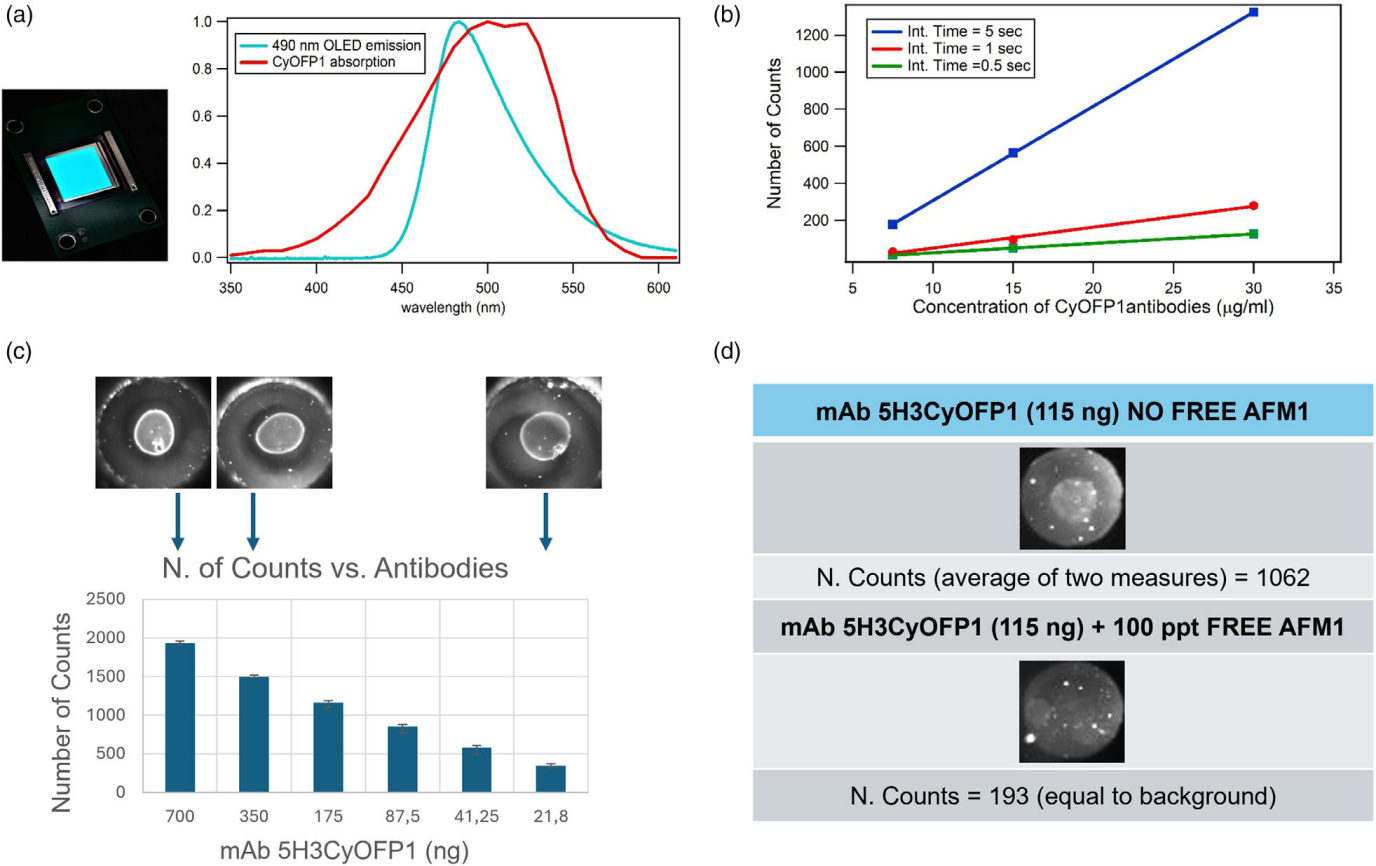
OLED‐based device setup. (a) Left: Image of the OLED with spectral emission at 490 nm; Right: normalized spectral emission of the 490 nm emitting OLED overlapped to the protein CyOFP1 normalized spectral absorption; (b) Fluorescence Intensity (in Counts) as a function of the 5H3CyOFP1 concentration (2 μL of different dilution deposited on a transparent plastic support) and image acquisition integration time. (c) Fluorescence signal (number of counts measured with a 12‐bit digital CCD camera), of different amounts of 5H3CyOFP1 after binding to BSA‐AFM1 deposited on the reaction chamber transparent substrate; on top of the graph, the fluorescence images of fluorescent complexes obtained at three different mAb concentrations. Values represent average ± SD (*n* = 2); (d) Result Preliminary competitive test: fluorescent images obtained with 115 ng of 5H3CyOFP1 bound to AFM1‐BSA deposited on the reaction chamber, without free AFM1 (top image) or adding 100 pg/mL of free AFM1 to the 5H3CyOFP1 solution (bottom image).

The images of the fluorescence emission obtained with a 2 μL drop of different dilutions of mAbs deposited on a transparent plastic support are shown in Figure S6 and the measured intensity (number of counts as an average of the illuminated pixels in the bright spots after the background subtraction) is also reported. The 5H3CyOFP1 showed a higher fluorescent signal than 5H3GFP, however the fluorescence intensity was still low and not intense enough to be useful for a direct competitive assay. To increase the fluorescence signal of the mAb fused with CyOFP1, a new OLED with a different spectral emission was tested. This new OLED was based on the Alq3 emitting molecule inserted in a strong microcavity to obtain a peak wavelength of 490 nm and a FWHM of 38 nm, and its overlapping with the spectral absorption of the CyOFP1 protein is optimized, as shown in Figure 5a.

After the OLED replacement, a new set of images was acquired and in this case, the fluorescence signal was significantly more intense. Being the fluorescence signal proportional to the integration time of the CCD used to acquire the fluorescence image, a set of images obtained with different dilutions of 5H3CyOFP1 was recorded and measured at different integration times. As shown in Figure 5a, the signals obtained with an integration time of 5 s have optimal intensity in relation to the amount of mAb spotted. In particular, the fluorescent signal obtained was a good compromise between spot visibility and the number of antibodies in the solution, also in view of the development of a direct competitive assay.

A preliminary study on the applicability of the antibody 5H3CyOFP1 for the development of a portable immunoassay for AFM1 quantification has been carried on. The transparent bottom part of a reaction chamber (Melpignano *et al*., 2016) was coated with the antigen AFM1‐BSA and different dilution of 5H3CyOFP1 in solution were incubated.

The fluorescent signals produced after the binding of the antibody to the antigen have been acquired using the POC OR‐EL, with the 490 nm emitting OLED device reader that presents a high‐sensitive CCD camera integrated with a microscope objective. All the measures were performed with an integration time of 5 s.

For the analysis, the background of each image has been subtracted, and the images obtained have been analyzed by applying a threshold to automatically identify and measure the circular fluorescent spots. To obtain the number of counts for each analyzed spot, the fluorescence intensity has been calculated by averaging the pixel values inside the image's bright spots' area, using the image processing software ImageJ. As shown in Figure 5c, the fluorescence signal followed an exponential behavior, and the fluorescence signal became not readable for a mAb amount of <21 ng.

Furthermore, a first competitive test was carried out using 5H3CyOFP1. In this assay, a quantity of antibody (approximately 100 ng) considered suitable for competition with free aflatoxin was used. The signal obtained with this amount of fluorescent antibodies was in the order of >1000 counts, which is a good compromise between spot visibility and the number of antibodies in the solution.

This starting fluorescent signal intensity is necessary to have an acceptable image contrast for the detection of the signal decrease due to the mAb competition with the free AFM1, to obtain a good assay sensitivity for the quantification of toxins as required by the European Community.

In the assay the 5H3CyOFP1 was incubated on the reaction chamber, with AFM1‐BSA deposited, without free AFM1 or adding 100 pg/mL of free AFM1.As can be seen in Figure 5d the fluorescence signal dropped significantly after the addition of the free AFM1, confirming the competition between the free AFM1 and the BSA‐AFM1 bounded to the reaction chamber substrate. While the immunoassay still needs significant optimization, the suitability of the 5H3CyOFP1 for the development of a direct competitive assay is confirmed.

### Conclusions

New diagnostic systems fast, sensible and easy to use for on‐site screening are increasingly in demand. To develop assays with these characteristics, in this work fluorescent antibodies were biotechnologically engineered by fusion to GFP or CyOFP1. These biomolecules could be used as ready‐to‐use reagents for different diagnostic systems. Biotechnological labelling has the dual advantage of obtaining a modified reagent with a single step of production and purification and ensuring constant sample homogeneity in all preparations with a 1:2 antibody/fluorophore ratio. Consequently, the diagnostic assay that will be developed will have great robustness and reproducibility and, if a primary antibody is labelled, the analysis time will also be advantageously reduced.

Both fluorescent antibodies were expressed in plants with good yields, although further optimization of the yield, especially of 5H3CyOFP1, can be expected in the future and they retained the affinity for the antigen, namely for the aflatoxin M1. After characterization, fluorescent Abs were used to set up two different fast screening systems: a LFIA and an OLED‐based device. Both recombinant antibodies proved to be good reagents for both diagnostic systems, giving an antigen‐specific fluorescence signal, although 5H3GFP was not used for further experiments exclusively for technical limitations (intrinsic fluorescence of nitrocellulose under UV illumination and non‐optimal excitation wavelength of OLED). A preliminary setup of the LFIA and OLED‐based device, as well as a first competitive assay for aflatoxin M1 detection using 5H3CyOFP1, was achieved, with good results. Furthermore, considering that both assays, based on new fluorescent antibodies, last only a few minutes, the possibility of developing a sensitive and economical test seems very promising.

## Experimental procedures

### Chemicals and materials

Sucrose, boric acid, sodium tetraborate decahydrate, bovine serum albumin (BSA), anti‐mouse immunoglobulin G antibody produced in rabbit (RAM) tris(hydroxymethyl)aminomethane, sodium dihydrogen phosphate monohydrate, sodium monohydrogen phosphate dihydrate, Tween 20 and sodium azide were obtained from Merck/Sigma–Aldrich (St. Louis, MO). The antigen AFM1‐BSA was synthesized as previously reported (Capodicasa *et al*., 2022).

Nitrocellulose (NC) membranes (HF180 plus card) and cellulose adsorbent pads were obtained from Merck Millipore (Billerica, MA). 2‐(N‐morpholino) ethanesulphonic acid was obtained from VWR International (Milan, Italy).

### Plant expression

#### Constructs for plant expression of fluorescent mAbs

The sequence encoding the Green Fluorescent Protein, optimized for expression in plants (mGFP5ER), was amplified by PCR with the AccuPrime™ Pfx DNA Polymerase (Invitrogen) from pBIN m‐gfp5‐ER (Haseloff *et al*., 1997), using the primer GCCGAATTCAGTAAAGGAGAAGAAC as forward and the primer CGATCTAGTAACATAGATGAC as reverse. The amplified DNA was purified with the PCR purification kit (Qiagen), digested with EcoRI/SacI, then inserted, by ligation, into an intermediate cloning vector, pBS‐Fc, containing the sequence encoding for a portion of the mouse immunoglobulin G1 heavy chain, digested with the same restriction enzymes. The pBS‐Fc was obtained by amplification of the heavy chain of 5H3 with primers CGCAACCCCGGGAGGAG as forward and CGGAATTCGGGACCAGGAGAGTGGGAG as reverse to permit the fusion of GFP at the C‐terminal of the heavy chain through the introduction of an EcoRI site at the end of the gene. The ligation product, purified by gel filtration chromatography, was used to transform *E. coli* XL1by electroporation. After a colony PCR screening using DreamTaq‐Green DNA Polymerase (Thermo Scientific), the plasmid DNA of two positive colonies was sequenced (BMR genomics). The DNA encoding the gene fusion was excised from pBS with the restriction enzymes XmaI and SacI and then inserted by ligation in pBI‐HC5H3 digested with Xma I/Sac I. *E. coli* XL1 cells were transformed by electroporation with the ligation and screened by PCR. The pBI‐HC5H3GFP plasmid obtained from a positive colony using the QIAprep Spin Miniprep Kit® (QIAgen) was then sequenced (BMR genomics) and employed for the transformation of *A. tumefaciens* LBA4404.

For 5H3CyOFP1, a synthetic sequence encoding the fluorescent protein CyOFP1 optimized for plant expression in *N. benthamiana* (Genescript) was designed and fused to the sequence encoding 5H3 heavy chain using the same strategy employed for 5H3GFP. Briefly, the gene was digested from the pUC57 plasmid with the restriction enzymes EcoRI and SacI and inserted, by ligation, into the pBS‐Fc. After electroporation of *E. coli* XL1, plasmid DNA extracted from a positive colony was digested with the restriction enzymes XmaI and SacI to excise and insert the fusion gene in the pBI. The pBI‐5H3HCCyOFP1, obtained as already described, was introduced by electroporation into *A. tumefaciens* LBA4404.

The construct used for light chain expression was the same already described in (Capodicasa *et al*., 2022) named pBI‐L5H3.

#### Infiltration of *Nicotiana benthamiana* plants with *A. tumefaciens*

To obtain full IgG, *A. tumefaciens* transformed with pBI‐HC5H3_fluorescent protein and pBI‐L5H3 were inoculated separately o/n at 28 °C, at 250 rpm, centrifuged at 4.000 × ***g*** and the pellet was resuspended in 10 mM MES, pH 5.6 and 10 mM MgCl_2_ to reach a final concentration of O.D. 600 nm = 0.6 of each culture. In addition, to enhance the expression levels of recombinant antibodies, agrobacteria harbouring the gene silencing suppressor p19 from TBSV (Danielson and Pezacki, 2013) were added to the mix (at 1:1:1 ratio). This agrobacteria suspension was infiltrated, using syringe or a vacuum chamber, into the intercellular spaces of 6‐week‐old *N. benthamiana* plant leaves. Plants were grown in peat pots weekly fertilized with nutrient solution (Supreme, Compo) diluted 1:300, at 24 °C and 70% humidity, under LED lamps (FLUXstrip 3500K, CreScience) (170 μmol/s/m^2^) with a 16 h light and 8 h dark cycle. Preliminary expression tests of the single constructs were, instead, carried out on a few plants, infiltrating the lower page of leaves manually, with a syringe without needle. To monitor the expression of the protein of interest, infiltrated leaf disks were sampled at days 4, 5, 6, 7 post‐infiltration frozen in liquid N2 and stored at −80 °C.

#### Western blot analysis

Total soluble leaf proteins (TSP) were extracted by grinding infiltrated leaves with pestle in eppendorf and their concentration was calculated by Bradford assay. Ten micrograms of TSP were separated by 10% sodium dodecyl sulphate–polyacrylamide gel electrophoresis (SDS‐PAGE) under reducing conditions and transferred onto a polyvinylidene fluoride sheet (PVDF: Immobilon‐P, Millipore) with a Semi‐dry Gel unit TE70X system (HOEFER). The PVDF membrane was blocked with 5% (w/v) skim milk in PBS (PBSM) 2 h at RT and incubated for 1 h at RT in 2% PBSM, containing the HRP‐labelled goat anti‐mouse IgG (KPL) diluted 1:5000. Subsequently, after washing (two in PBS‐Tween 0.1% and two in PBS) the membrane was developed with a chemiluminescent protein detection solution (Immobilon Substrate, Millipore). The signal was then detected using an image acquisition system (iBright Thermofisher).

### Purification and characterization of recombinant fluorescent mAbs

#### Fluorescent abs purification

Leaves of vacuum‐agroinfiltrated plants, harvested at 6 dpi were grinded with liquid nitrogen in mortars; the powder obtained was resuspended with PBS (two volumes of plant material weight) and homogenized by ultraturrax (IKA). Subsequently, the homogenate was pre‐filtered on Miracloth (Sigma‐Aldrich) and centrifuged at 40 000 × ***g*** for 30 min at 4 °C. The supernatant was then further filtered with a 0.22 μm membrane (PVDF, Millipore) and loaded on a 1 mL HiTrapTM Protein G HP column (Cytiva). Since protein G has high affinity for IgG at pH 7, the column was preliminarily equilibrated with PBS (10 column volumes), through a loading flow of 1 mL/min with a peristaltic pump. The column was washed with additional 10 volumes of PBS buffer, to remove unbound material. For elution was used 100 mM glycine buffer, pH 2.7 (5 column volumes), immediately neutralized with 1 M Tris–HCl (pH 9). Eluted fractions were analysed by 10% SDS‐PAGE. Collected fractions containing antibodies were dialysed and concentrated by ultrafiltration, using Amicon Ultra 50 K centrifugal filter (Millipore) and the antibody concentration was determined by spectrophotometry.

#### Size‐exclusion chromatography

The purified recombinant Abs were analysed by size‐exclusion chromatography at 20 °C in PBS on a Superdex™ 75 10/300 GL column (GE Healthcare) (flow rate, 0.4 mL/min) using an AKTA FPLC P920 instrument (GE Healthcare). Protein absorbance, expressed as absorption units (mAU), was measured at 280 nm. Column calibration was performed using gel filtration calibration kits (low and high molecular weight, GE Healthcare), following the manufacturer's instructions. Purified 5H3 mAb and mouse IgG (I8765, Sigma‐Aldrich) were also used as controls.

#### SDS‐page

Purified fluorescent mAbs were resolved on 10% sodium dodecyl sulfate‐polyacrylamide gel electrophoresis (SDS‐PAGE) in reducing condition or 4%–12% Tris‐glycine gradient SDS‐PAGE in non‐reducing condition. The gel was stained with Coomassie blue or the fluorescence signal was acquired (exc = 490–520 nm, em = 568–617 nm) by iBright FL1500 (Thermofisher).

#### ELISA

The quantification of extracts and the binding kinetics of purified antibodies were analysed by ELISA assay. Maxisorp ELISA plates (Nunc) were coated with 100 μL/well AFM1‐BSA (1 ng/μL, Sigma) in PBS, overnight at 4 °C. Plates were washed 4 times with 300 μL/well washing buffer (PBS with 0.05% v/v Tween 20) and blocked for 2 h at 37 °C with 200 μL of BSA 1%. Plates were washed again and 100 μL/well of variable amounts of plant extracts in PBS‐BSA 0.2% were added, and incubated for 1 h at 37 °C. For the quantification of recombinant proteins in the extracts, the absorbance signals were compared with the signals obtained by a reference curve produced with known concentrations of purified mAb 5H3 (from 80 to 5 ng/well). After incubation, plates were washed and then 100 μL/well of HRP‐labelled goat anti‐mouse IgG (γ) (KPL) was applied at 1:2000 in PBS‐BSA 0.2%, and incubated for 1 h at 37 °C. After an additional 4 washing steps, the signal was developed with 100 μL of TMB or ABTS. The absorbance was measured after 15 min at 450 nm for the TMB substrate, after chemical stop of the reaction with acid solution (3 M HCl) and at 405 nm for the ABTS substrate, using an ELISA reader (F50, TECAN). For binding kinetics evaluation, serial dilutions (1:1) of equimolar concentrations of the antibodies were tested (starting from 13.33 to 0.21 nM).

To evaluate the competition with the free toxin, an indirect competitive ELISA (icELISA) was carried out. The same coating conditions were applied, modifying the reaction volume to 200 ul/well. A pre‐incubation step, between the antibodies and free aflatoxin M1, of 45 min at RT was performed, mixing a normalized quantity of recombinant antibodies (15 ng of 5H3 and about 20 ng of the fluorescent antibodies) with variable concentrations of purified aflatoxin M1 (from 400 to 12.5 ng/L).

The binding parameters were then obtained using a Langmuir binding isotherm model:

$$S=\left(S_{\max}\times x\right)/\left(k_{d}+x\right)$$

where *x* is the amount of the mAb, *S* is the signal measured and K_d_ is the apparent dissociation constant and *S*_max_ represents the signal at the saturation of binding sites.

Competition curves were fitted by four‐parameter logistic equation (Wild, 2013), where y was B/B_0_ (the signal normalized for the signal of the blank) and *x* was the AFM1 concentration; max and min are the values of the lower and upper asymptotes, respectively, slope is the slope at the inflection point and IC50 corresponds to the analyte concentration that inhibited the maximum signal at 50%. Statistical comparison of IC50 values was performed by the One‐Way ANOVA (α = 0.05). Fitting and statistical analysis were performed by the software SigmaPlot 12.0 (Grafiti, CA).

### LFIA development

#### Production of the LFIA dipstick devices

The NC membrane used for the initial assessment of recombinant fluorescent mAbs comprised one reactive line obtained by anchoring the RAM antibody as the capturing reagent (1 mg/mL). The LFIA device was prepared by anchoring the AFM1‐BSA bioconjugate (0.5 mg/mL) and the RAM antibody (1 mg/mL) onto the NC membrane to form the test and control lines respectively. Reagents were dotted at 1 μL × cm^−1^ by means of a XYZ3050 platform (Biodot, Irvine, CA), equipped with BioJetQuanti TM 3000Line Dispenser for non‐contact deposition. NC membranes were dried at 37 °C for 60 min under vacuum. A cellulose adsorbent pad was overlayed to the NC membrane, with 1‐2‐mm overlapping. The membranes were then cut into strips (3 mm width) by means of a CM4000 guillotine (Biodot, Irvine, CA) and stored in plastic bags at room temperature until use.

#### Applicability of the 5H3GFP and 5H3CyoFP1 as probes for LFIA

The composition of the diluent buffer was defined by comparing three buffers: MES buffer (20 mM pH5), phosphate buffer (20 mM pH7.4) and Tris–HCl buffer (20 mM pH8.5), and by varying the concentration of Tween20 (0%–0.5%–1.0%–2.0% v/v) and BSA (0%–0.5%–1% w/v) added. Membranes were dipped into the well of a microtiter plate, containing 5H3GFP (500 ng/well) diluted with 70 μL of the various buffers and flowed for 15 min. The intensity of the fluorescence localized in correspondence with the test line was measured after excitation at 366 nm by means of the CAMAG UV cabinet 4 (Camag, Muttenz, Switzerland). The images were collected by smartphone and analyzed using a free image processing software (ImageJ 1.53e, National Institutes of Health, United States).

The 5H3CyOFP1 probe was applied to a LFIA strips including AFM1‐BSA as the specific antigen (test line). The probe was added to a microplate well containing 70 μL of the dilution buffer (Tris–HCl 20 mM pH 8.5 supplemented with 0.5% Tween 20 and 1%BSA). The solution was flowed for 15 min, and the fluorescence signal was recorded by iBright FL1500 (exc = 490–520 nm em = 568–617 nm) (Thermofisher). Collected images were analyzed using the ImageJ processing software to quantify the emitted light.

For both systems, the dependency of the fluorescent signal on the concentration of the recombinant fluorescent mAbs was investigated using serial dilution of the probe by a factor of 2, from 600 ng/well. The effect of increasing the ionic strength (80–130–280–480 mM of NaCl) and varying the pH (5, 7.4 and 8.5) of the diluent buffer were studied by diluting 300 ng/well of the probe in the proper solution.

The reproducibility of signal obtained by the 5H3CyOFP1‐based LFIA was assessed by repeating measurements twice on each day for 3 days and for 2 levels of 5H3CyOFP1 (high: 300 ng/well and low: 37.5 ng/well). The intra‐day coefficient of variability (CV%) was calculated as the average of the CV% calculated for the 2 replicates obtained on each day. The inter‐day CV% was calculated from the mean signals obtained for each level on the 3 different days.

The specificity of the 5H3CyOFP1 binding to the antigen was verified by adding AFM1 (2 ng/well) to the solution that contained 150 ng/well of the 5H3CyOFP1 and observing the decrease of the test line fluorescence.

### OLED‐based device development

#### Applicability of the 5H3GFP and 5H3CyOFP1 as probes for OLED‐based diagnostic device

The fluorescence intensities of 5H3GFP and 5H3CyOFP1 were measured in transmission using a plastic substrate, with optical transmission of >90% in the visible range, in which a drop of 2 μL of different dilutions of mAbs was deposited. A high sensitivity CCD camera (described above) was used to record the fluorescence images and after the background subtraction, the fluorescence intensity was calculated as the average of the illuminated pixels in the circular spot using the image processing software ImageJ.

For immunoassay, the polyethylene (PE) transparent bottom part of the reaction chamber was coated with a drop of 1.5 μL of BSA‐AFM1 (1 mg/mL) mixed with Carbonate buffer 0.05 M (pH 9.6) at a ratio of 1:5 and incubated overnight at RT. Subsequently, the cartridges were washed twice with PBS‐Tween 20 (0.05%) to remove unbonded BSA‐AFM1, and a blocking step was performed with a protein‐based solution at pH 7.4 for 1 h at 37 °C. Then, after the washing step, the immunoassay reaction was performed, incubating at 40 °C for 10 min different concentrations of 5H3CyOFP1, 35 μL of serial dilution 1:1 starting from 700 ng of mAb in PBS, in the reaction chamber of the cartridge. Following two washing steps with PBS‐Tween 20 (0.05%), the cartridge was left to air dry before the fluorescence detection. The measures have been performed in duplicate for every mAb concentration.

The preliminary competition test was carried out incubating 115 ng of 5H3CyOFP1 in the reaction chamber of a cartridge, coated with AFM1_BSA as already described, in the presence or absence of free AFM1, at a concentration of 100 pg/mL.

#### Organic light emitting diodes (OLED) used

The first OLED used in this experiment has a peak wavelength of 434 nm and a FWHM of about 70 nm, it is powered at constant current of 20 mA giving a radiometric luminance of 300 μW/cm^2^. Its molecular architecture is based on the emission of the hole‐transport molecule α‐NPD [N,N0‐di‐1‐naphthyl‐N,N0‐diphenyl‐1,10‐biphenyl4,40‐diamine] in a weak microcavity, as described in Marcello *et al*., 2013. The second OLED, optimized for the fluorescence excitation of the protein CyOFP1 is based on the emission of the electron transport and emitting molecule Alq3 [Tris(8‐hydroxyquinoline)aluminium(III)] inserted in a strong microcavity to blue‐shift its standard peak emission in order to obtain a peak wavelength of 490 nm and a FWHM of 38 nm. It was powered at a constant current of 15 mA giving a radiometric luminance of 250 μW/cm^2^ (Patent EP2491371B1 and US 8647578B2).

#### Fluorescence acquisition optical setup

For these all experiments, a high sensitivity CCD camera was used for the image acquisition (Hamamatsu C8484‐03G02) with 12‐bit digitalization corresponding to a signal range from 0 (dark) to 4095 (saturation). The images were recorded using a binning of 8 and no gain. The integration time was set at 20 s for the images recorded using the first OLED, while different integration times were tested for the acquisition of the images of the CyOFP1 fused mAbs when using the second OLED for the fluorescence excitation. To select only the fluorescence emission and reduce the background, two different bandpass filters were used corresponding to the fluorescence peak wavelength of the two fluorescent proteins; in particular, for the detection of the CyOFP1 fluorescence, a bandpass filter centred at 607 nm with a bandpass of 36 nm (T > 90%) and extinction O.D. >5 was used, while for the GFP, a bandpass filter centred at 520 nm with a bandpass of 36 nm (T > 90%) and extinction O.D. >5 was selected.

## Conflicts of interest

The authors have no conflict of interest to declare.

## Author contributions

R.S. and M.C. performed experiments of gene engineering, plant expression and characterization of fluorescent Abs. T.S. and L.A. performed experiments and drafted figures and text of LFIA. P.M. performed experiments and drafted figures and text of OLED biosensor. C.C. designed and conducted the research and wrote the manuscript. All authors have read and agreed to the published version of the manuscript.

## Supporting information


**Figure S1** Recombinant fluorescent mAbs purification. Eluted fractions from affinity chromatography on protein G UV illuminated (a) and analysed by SDS‐PAGE in reducing conditions (b). Samples loaded: Precision Plus Protein™ Dual Colour Standards (Bio‐Rad), 10 μL (15 μg) of infiltrated plant extract loaded on column (Extr), 10 μL of column flowthrough (FT) and wash (W), 5 μL of eluted fractions (F), 2 μL of concentrated 5H3GFP (C), 1.5 μg of mAb 5H3 (mAb).

**Figure S2** Gel filtration analysis of 5H3GFP and 5H3CyOFP1. Chromatograms obtained by size‐exclusion chromatography on a Superdex 75 10/300 GL column of fluorescent and original 5H3 mAb (a) and of a set of low molecular weight protein standards, ranging from Mr 6500–75 000 and Blue dextrane in red (b). The retention volumes (mL) of the major peaks obtained are reported.

**Figure S3** Optimization of 5H3GFP fluorescent signals on LFIA strips. (a) Investigation of the 5H3GFP flow across the NC membrane functionalized with a reactive line of RAM antibodies: as an example, the effect of three buffer compositions is shown, resulting in no observable fluorescent signal at the RAM line (left), low (middle) and bright (right) fluorescent signals, indicating that the 5H3GFP did not flow, partially flowed and optimally flowed across the membrane, respectively. Luminescence signals as a result of the RAM‐5H3GFP complex formation at the reactive line were measured and plotted as a function of 5H3GFP probe dilution (b); of the ionic strength (c); and of the pH of the running buffer (d).

**Figure S4** Optimization of 5H3CyOFP1 fluorescent signals on LFIA strips. Normalized luminescence signals measured at the Test line as a function of the ionic strength (a); the pH of the running buffer (b). The signals at the Test line were produced by the selective binding of the AFM1‐5H3CyoFP1 to the antigen immobilized on the strip membrane.

**Figure S5** Competitive LFIA. B/N images of the 5H3CyOFP1‐based LFIA strips upon addition of samples containing no AFM1 (left) and 2 ng/well of AFM1 (right).

**Figure S6** Fluorescent antibodies tested in a standard OLED. Image (a) OLED with peak spectral emission at 434 nm; (b) Normalized spectral emission of the 434 nm emitting OLED overlapped to the protein CyOFP1 normalized spectral absorption; (c) Normalized spectral emission of the 434 nm emitting OLED overlapped to the protein GFP normalized spectral absorption; (d) Fluorescence intensity of different amounts of 5H3CyOFP1 excited with the 490 nm emitting OLED and 5H3GFP excited with the 434 nm emitting OLED (2 μL drops); (e) Fluorescence Intensity (in Counts) depending on antibody concentration and image acquisition integration time.

**Table S1** Reproducibility of signals provided by the LFIA device including 5H3CyOFP1.

## Data Availability

The data that support the findings of this study are available on request from the corresponding author. The data are not publicly available due to privacy or ethical restrictions.
