# Supplementary figures and images for: Generation and plant production of recombinant fluorescent immunoglobulin G as innovative immunodiagnostic reagents
Source: Plant Biotechnol J. 2025 Jul 1;24(1):300–12. doi: 10.1111/pbi.70235 (PMC12854897; doi:10.1111/pbi.70235)

A

5H3GFP

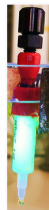

5H3CyOFP1

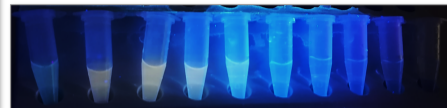

Fract. 1 2 3 4 5 6 7 8 9 10

B

M Extr FT W F2 F4 F6 F8 C mAb

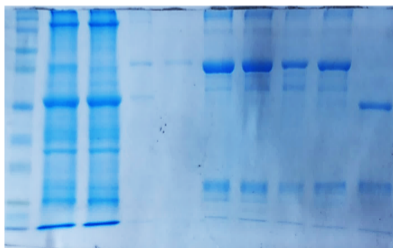

← 76 kDa

← 50 kDa

← 25 kDa

M Extr FT W F1 F2 F3 F4 F5 F6

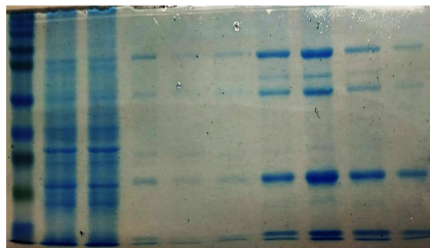

← 77 kDa

← 50 kDa

← 25 kDa

Supplement: Supplementary file 1 — Figure S1 Recombinant fluorescent mAbs purification. Eluted fractions from affinity chromatography on protein G UV illuminated (a) and analysed by SDS‐PAGE in reducing conditions (b). Samples loaded: Precision Plus Protein™ Dual Colour Standards (Bio‐Rad), 10 μL (15 μg) of infiltrated plant extract loaded on column (Extr), 10 μL of column flowthrough (FT) and wash (W), 5 μL of eluted fractions (F), 2 μL of concentrated 5H3GFP (C), 1.5 μg of mAb 5H3 (mAb). [file PBI-24-300-s001.pdf]

**A**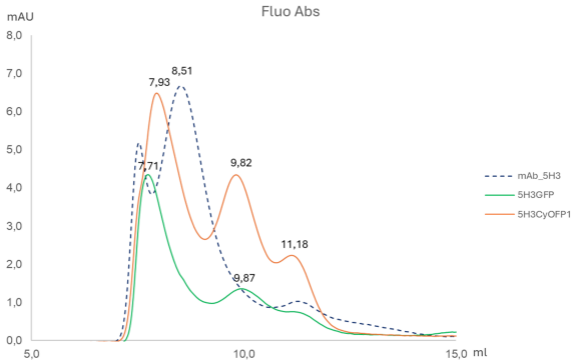**B**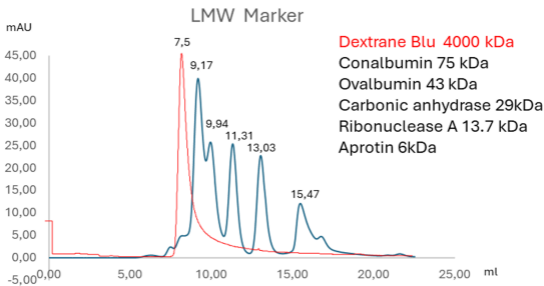

Supplement: Supplementary file 2 — Figure S2 Gel filtration analysis of 5H3GFP and 5H3CyOFP1. Chromatograms obtained by size‐exclusion chromatography on a Superdex 75 10/300 GL column of fluorescent and original 5H3 mAb (a) and of a set of low molecular weight protein standards, ranging from Mr 6500–75 000 and Blue dextrane in red (b). The retention volumes (mL) of the major peaks obtained are reported. [file PBI-24-300-s007.pdf]

**A**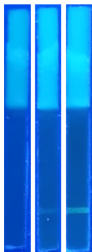**B**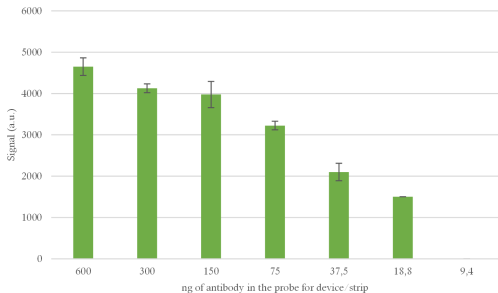**C**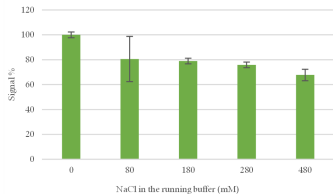**D**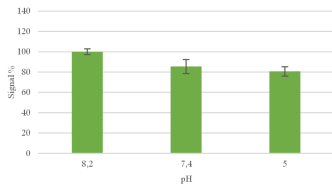

Supplement: Supplementary file 3 — Figure S3 Optimization of 5H3GFP fluorescent signals on LFIA strips. (a) Investigation of the 5H3GFP flow across the NC membrane functionalized with a reactive line of RAM antibodies: as an example, the effect of three buffer compositions is shown, resulting in no observable fluorescent signal at the RAM line (left), low (middle) and bright (right) fluorescent signals, indicating that the 5H3GFP did not flow, partially flowed and optimally flowed across the membrane, respectively. Luminescence signals as a result of the RAM‐5H3GFP complex formation at the reactive line were measured and plotted as a function of 5H3GFP probe dilution (b); of the ionic strength (c); and of the pH of the running buffer (d). [file PBI-24-300-s006.pdf]

**A**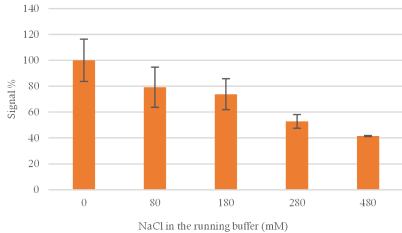**B**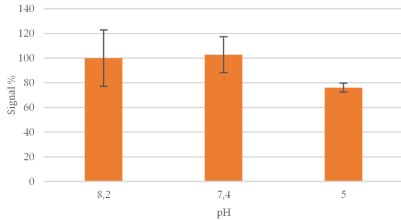

Supplement: Supplementary file 4 — Figure S4 Optimization of 5H3CyOFP1 fluorescent signals on LFIA strips. Normalized luminescence signals measured at the Test line as a function of the ionic strength (a); the pH of the running buffer (b). The signals at the Test line were produced by the selective binding of the AFM1‐5H3CyoFP1 to the antigen immobilized on the strip membrane. [file PBI-24-300-s005.pdf]

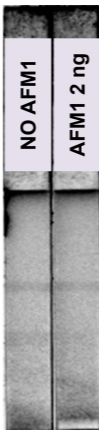

← Control line  
(RAM)

← Test line  
(AFM1-BSA)

Supplement: Supplementary file 5 — Figure S5 Competitive LFIA. B/N images of the 5H3CyOFP1‐based LFIA strips upon addition of samples containing no AFM1 (left) and 2 ng/well of AFM1 (right). [file PBI-24-300-s003.pdf]
