# Supplementary material for: Generation and plant production of recombinant fluorescent immunoglobulin G as innovative immunodiagnostic reagents
Source: Plant Biotechnol J. 2025 Jul 1;24(1):300–12. doi: 10.1111/pbi.70235 (PMC12854897; doi:10.1111/pbi.70235)
Supplement: Supplementary file 6 — Figure S6 Fluorescent antibodies tested in a standard OLED. Image (a) OLED with peak spectral emission at 434 nm; (b) Normalized spectral emission of the 434 nm emitting OLED overlapped to the protein CyOFP1 normalized spectral absorption; (c) Normalized spectral emission of the 434 nm emitting OLED overlapped to the protein GFP normalized spectral absorption; (d) Fluorescence intensity of different amounts of 5H3CyOFP1 excited with the 490 nm emitting OLED and 5H3GFP excited with the 434 nm emitting OLED (2 μL drops); (e) Fluorescence Intensity (in Counts) depending on antibody concentration and image acquisition integration time. [file PBI-24-300-s004.pdf]

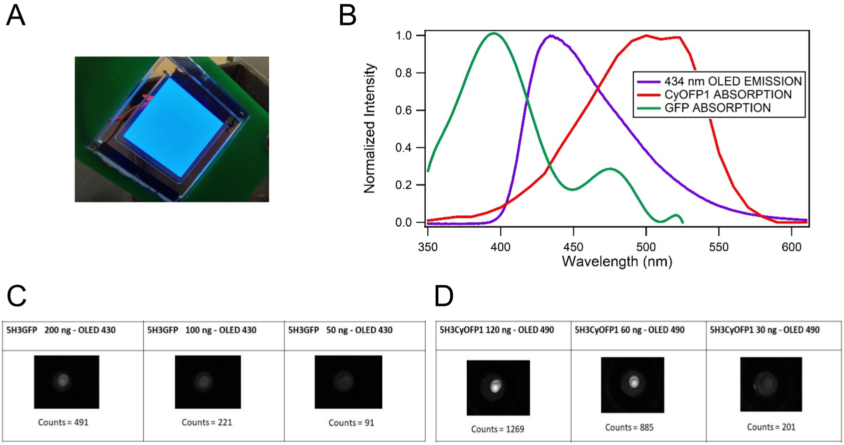
A
B
1.0
0.8
0.6
0.4
0.2
0.0
Normalized Intensity
350
400
450
500
550
600
Wavelength (nm)
434 nm OLED EMISSION
CyOFP1 ABSORPTION
GFP ABSORPTION
C
5H3GFP 200 ng - OLED 430
Counts = 491
5H3GFP 100 ng - OLED 430
Counts = 221
5H3GFP 50 ng - OLED 430
Counts = 91
D
5H3CyOFP1 120 ng - OLED 490
Counts = 1269
5H3CyOFP1 60 ng - OLED 490
Counts = 885
5H3CyOFP1 30 ng - OLED 490
Counts = 201
