# Supplementary material for: Generation and plant production of recombinant fluorescent immunoglobulin G as innovative immunodiagnostic reagents
Source: Plant Biotechnol J. 2025 Jul 1;24(1):300–12. doi: 10.1111/pbi.70235 (PMC12854897; doi:10.1111/pbi.70235)
Supplement: Supplementary file 7 — Table S1 Reproducibility of signals provided by the LFIA device including 5H3CyOFP1. [file PBI-24-300-s002.docx]

**Table S1: Reproducibility of signals provided by the LFIA device including 5H3CyOFP1.**

|  | 5H3CyOFP1 | |
| --- | --- | --- |
|  | high | low |
| CV% intra-day (n=2x3=6) | 8.4 % | 11.1 % |
| CV% inter-day (n=3) | 9.7 % | 10.6 % |

Two levels of the probe were studied: high (300 ng/strip) and low (37,5 ng/strip). CV% were calculated as the means of two replicate measurements carried out on each day for three days (intra-day) and as the coefficient of variation of three replicate measurements carried out on three days (inter-day)
